# Effect of Diamond Burnishing on Fatigue Behaviour of AISI 304 Chromium-Nickel Austenitic Stainless Steel

**DOI:** 10.3390/ma15144768

**Published:** 2022-07-07

**Authors:** Jordan Maximov, Galya Duncheva, Angel Anchev, Vladimir Dunchev, Yaroslav Argirov

**Affiliations:** 1 Department of Material Science and Mechanics of Materials, Technical University of Gabrovo, 5300 Gabrovo, Bulgaria; duncheva@tugab.bg (G.D.); anchev@tugab.bg (A.A.); v.dunchev@tugab.bg (V.D.); 2 Department of Material Sciences, Technical University of Varna, 9010 Varna, Bulgaria; jaroslav.1955@abv.bg

**Keywords:** austenitic stainless steels, enhancement of fatigue strength, diamond burnishing, residual stresses, strain-induced martensite

## Abstract

The disadvantages of widely used austenitic stainless steels are their low hardness and relatively low fatigue strength. Conventional chemical-thermal surface treatments are unsuitable for these steels since they create conditions for inter-granular corrosion. An effective alternative is a low-temperature surface treatment, creating an S-phase within the surface layer, but it has a high cost/quality ratio. Austenitic steels can increase their surface micro-hardness and fatigue strength via surface cold working. When the goal is to increase the rotating bending fatigue strength of austenitic chromium-nickel steels, and the requirements for significant wear resistance are not paramount, diamond burnishing (DB) has significant potential to increase the fatigue strength and, based on the cost/quality ratio, can successfully compete with low-temperature chemical-thermal treatments. The main objective of this study is to establish the effect of DB on the rotating fatigue strength of AISI 304 L chromium-nickel austenitic steel. The influence of DB parameters on the surface integrity (SI) characteristics was studied. Optimal DB parameters under minimum roughness and maximum micro-hardness criteria were obtained. Rotating bending fatigue tests of the diamond burnished (in a different manner) and untreated specimens were performed. DB implemented via parameters providing maximum micro-hardness increased fatigue limit by 38% compared to untreated specimens.

## 1. Introduction

Stainless steels are resistant to atmospheric, soil, acid, basic and other electrochemical corrosion processes, as well as seawater. After introducing more than 13% chromium, the electrochemical potential of steel is positive, and it acquires resistance to corrosion in atmospheric and other conditions. Stainless steels are divided into the following classes depending on their structure: martensitic, martensitic-ferritic (ferrite > 10%), ferritic, austenitic-ferritic, austenitic and austenitic-martensitic. Austenitic steels are the most important class in terms of relative share and universality of use and account for 70% of world production of all types of stainless steels. According to their chemical composition, these steels are divided into chromium-nickel and chromium-manganese, and the relative share of the first type is significantly higher. The object of the present study is AISI 304 austenitic chromium-nickel steel. This steel is used in many industrial applications as a structural component, owing to its superior corrosion resistance, excellent formability, good machinability by cutting and weldability. In addition, it is among the cheapest grades of austenitic stainless steels, making it a favourite choice in industry. However, its main disadvantages which can limit its applications are the low surface micro-hardness, which leads to low wear resistance and relatively low strength, including fatigue strength. Furthermore, the temperature interval $500–700{\;}^{{}^{\circ}}C$ is very dangerous for chromium-nickel stainless steels as it creates conditions for intergranular corrosion due to the release of chromium carbides at the boundaries of the austenitic grains. Therefore, the conventional chemical-thermal surface treatments (i.e., nitriding, carburising, cyanidation) can achieve a short-term effect by increasing the surface hardness and hence the fatigue strength in rotating bending, as this effect is at the expense of the reduced corrosion resistance.

Effective alternatives are the so-called low-temperature surface treatments, which are performed with nitrogen- and/or carbon-containing media at temperatures below $500{\;}^{{}^{\circ}}C$ in which the mobility of chromium is low and the formation of precipitates is hindered [1]. These treatments lead to the obtaining of the so-called S-phase (or expanded austenite, m-phase, $\gamma_{N}$, $\gamma_{C}$) in the surface layer, which is characterised by high micro-hardness and residual compressive stresses up to (2–3) GPa. As a result, the wear resistance and fatigue strength increase dramatically. In recent years, many studies have been carried out upon low-temperature nitriding [2,3,4,5,6,7] and carburisation [8,9,10,11], to confirm the beneficial properties of the S-phase. A detailed analysis and bibliographic reference on this subject are contained in the remarkable review paper by Francesca Borgioli [1].

Except by changing the chemical composition of the surface layers, chromium-nickel austenitic steels can increase their surface micro-hardness and strength, including fatigue strength, through cold working. The nickel reduces the ability of austenite to strain hardening, thus for steels that require a high degree of hardening, the nickel content is limited to 8–9%. At a higher degree of plastic deformation, the metastable austenite undergoes martensitic transformation. If the nickel content in the steel is greater than 15%, austenite is stable and in cold working—even with a high degree of plastic deformation—the formation of strain-induced martensite is not observed. The cold working causes the formation of two phases of strain-induced martensite: (1) body-centered-cubic ferromagnetic or tetragonal α’-martensite; (2) hexagonal-closed-packed ε-martensite. The second phase is formed at smaller deformations and, with increasing degree of plastic deformation, is transformed into α’-martensite [12,13]. Numerous studies have been devoted to changing the properties of chromium-nickel stainless steel by volumetric cold plastic deformation, mainly through cold rolling. Mitra et al. [14] studied the ferromagnetic properties of plastically deformed by cold rolling AISI 304 steel sheets (martensite volume fraction below 58%) using magnetic hysteresis and Barkhausen emission methods. Hedayati et al. [15] investigated the effect of strain-induced α’-martensite on the microstructure and mechanical properties of cold-rolled AISI 304 L steel sheets. The authors showed that the formation of strain-induced martensite clearly resulted in significant steel strengthening. Kim et al. [16] studied the effect of solution annealing on the α’-martensitic microstructure of cold rolled (10% and 20%) AISI 316 L steel. The authors established that solution annealing and α’-martensitic microstructure played an important role in stress corrosion cracking resistance of this steel. The evolution of texture during cold rolling (up to 90% deformation) of AISI 304 steel was conducted by Kumar et al. [17]. The authors established that the texture in martensite is controlled from the initial texture of the main austenitic phase even to maximum levels of plastic deformation. The influence of cold plastic deformation in the range of 18–79%, introduced by the cold rolling process, on the microstructure and mechanical properties of X5CrNi18-8 (AISI 304) steel was studied by Kurc-Lisiecka and Kalinowska-Ozgowicz [18]. A similar study, but for nitrogen-bearing AISI 304N steel, was carried out by Li et al. [19]. The authors showed that the formation of α’-martensite and the lamellar grains resulted in the significant strengthening of the steel and a substantial decrease in elongation. Nanostructuring of the AISI 316 L specimen was achieved by Chen et al. [20] using a surface mechanical attrition treatment. The specimen yield limit was increased to 1450 MPa. The effect of different degrees of plastic deformation introduced by cold rolling upon the hardness and tensile properties of AISI 304 steel was investigated by Milad et al. [21]. Similar studies on this steel have been conducted by Singh et al. [22]. The authors established that the improvement in ultimate tensile strength of cold-rolled samples was due to the combined effect of grain refinement and stress-induced martensitic phase transformation. Suyitno et al. [23] studied the effect of cold plastic deformation (compressive strains in the range of 17–47%) and sandblasting on the micro-hardness, tensile strength and corrosion rate of AISI 316 L steel. They found that cold working improved the mechanical properties and corrosion resistance, while subsequent sandblasting impaired corrosion resistance. The impact of different levels of cold rolling (from 0 to 50%), respectively, on the amount of strain-induced ferromagnetic α′-martensitic phase, on the microstructure, magnetism, mechanical properties and corrosion behaviour of AISI 316 L steel was studied by Tanhaei et al. [24]. Tavares et al. [25] researched phase transformation in AISI 304 L steel. They established that the cold rolling induced α’-martensite, while the next high-pressure torsion promoted partial transformation from α’ to ε-phase.

While the effect of cold volumetric plastic deformation (and the phase transformations provoked by the cold working) on the mechanical properties of chromium-nickel austenitic steels has been widely studied, significantly less attention has been paid to increasing the fatigue strength and wear resistance through mechanical static surface treatment—so-called burnishing methods, regardless of their advantages: (1) significantly improve surface integrity (low values of height parameters of roughness, the favorable combination of values of roughness shape parameters, high micro-hardness, significant residual compressive stresses), while the core of the component remains tough; (2) the process is very economical (it is a green technology), low spare part consumption and saves time, money and energy; (3) long tool life and no operator skill is required; (4) machining time is short. The ability of chromium-nickel austenitic steels for strain hardening allows an increase in the surface micro-hardness and rotating bending fatigue strength via severe surface plastic deformation. Juijerm and Altenberger [26] implemented Ecoroll’s deep rolling process using a hydrostatic ball to increase the fatigue strength of AISI 304 steel. They achieved an increase of approximately 18% at $10^{6}$ cycles fatigue strength, from 280 to 330 MPa. Using single-pass diamond burnishing (DB), Maximov et al. [27] increased the fatigue limit ($10^{7}$ cycles fatigue strength) of AISI 316 Ti steel from 270 to 350 MPa, i.e., a rise of 29.6%. Applying four-pass DB, an increase of 38.9% is from 270 to 375 MPa. In addition, DB was implemented with process parameters, which were optimal under minimum roughness $R_{a}$ criteria, and not under maximum fatigue strength. When the goal is to increase the fatigue strength of chromium-nickel austenitic steels, DB is an excellent option being much cheaper and faster than low-temperature nitriding and carburising. For instance, Thaiwatthana et al. [28] established that both low-temperature-plasma nitrided (400 °C, 15 h) and carburised (415 °C, 12 h) AISI 316 steel specimens had an improved fatigue limit by more than 25% than untreated steel. It has been observed that carburised specimens have a higher fatigue limit than nitrided specimens because nitrided layers are harder but more brittle than carburised ones. Hoshiyama et al. [29] subjected to bending fatigue plasma-nitrided specimens made of AISI 304 steel treated at 400 °C for different durations (2–8 h). They found that fatigue strength tended to reach 449 MPa for a treatment duration of more than four hours. The increase compared to untreated samples is approximately 15%, from 390 MPa judging by (Figure 8) [29] to 449 MPa. Ceschini and Minak [30] showed that AISI 316 L steel specimens treated by low temperature carburising ($450{\;}^{{}^{\circ}}C$ for 100 h) and subjected to rotating bending fatigue, the fatigue limit (10^7^ cycles fatigue strength) reached 521 MPa, an increase of more than 40% compared to untreated steel (366 MPa). An additional increase in fatigue strength of up to 624 MPa can be obtained for carburised air-cooled specimens. From the above examples, it is clear that when the goal is to increase the rotating bending fatigue strength of austenitic chromium-nickel steels, and the requirements for significant wear resistance are not paramount, DB has a significant potential to increase the fatigue strength and, based on the cost-quality ratio, can successfully compete with low-temperature chemical-thermal treatments. Thus, the main objective of this study is to establish the effect of DB on the rotating fatigue strength of AISI 304 chromium-nickel austenitic steel.

## 2. Materials and Methods

### 2.1. Material

The chromium-nickel AISI 304 austenitic stainless steel was obtained as a cylindrical bar and underwent chemical analysis, mechanical testing, phase- and micro-structural analyses by the authors in our Testing of Metals Laboratory. Table 1 shows the chemical composition. Optical Emission Spectrometer (model Foundry-Master Optimum), manufactured by HITACHI, was used to determine the chemical composition. The apparatus measures the content in weight percentages, as the minimum value of the step for determining the content of a chemical element is 0.001.

Based on the chemical analysis, the nickel and chromium equivalents were calculated according to [31]: $Cr_{eq}=18.83\%$, $Ni_{eq}=10.22\%$. As the nickel equivalent is significantly less than 15%, a martensitic transformation due to DB can be expected.

**Figure 1:**
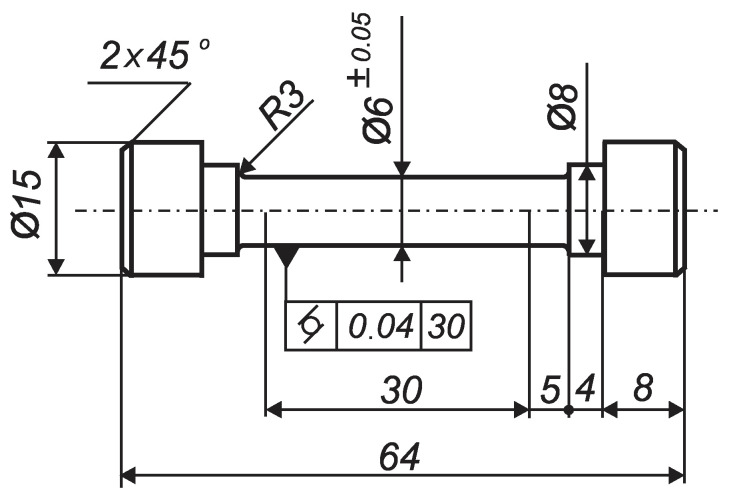
Tensile test specimen geometry (the sizes are in mm).

The essential mechanical characteristics of the steel in a state as received were established at room temperature using a Zwick/Roell Vibrophore 100 testing machine. The sizes of the specimens used (according to [32]) are shown in Figure 1.

Table 2 shows the arithmetic mean values of the mechanical characteristics obtained from three specimens. The maximum deviations do not exceed 2%.

**Figure 2:**
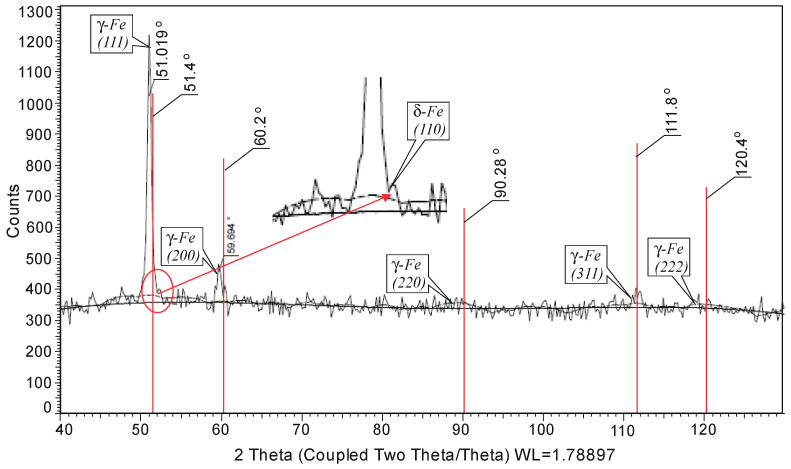
Phase analysis outcomes for AISI 304 steel.

The phase analysis was performed using a Bruker D8 Advance X-ray diffractometer. Crystallography Open Database was used to determine the peak positions. A shift of the maxima to the smaller angles is observed due to the deformation of the crystal lattice by the chromium and manganese dissolved in it. The X-ray diffraction pattern also shows the presence of residual δ-ferrite (Figure 2).

**Figure 3:**
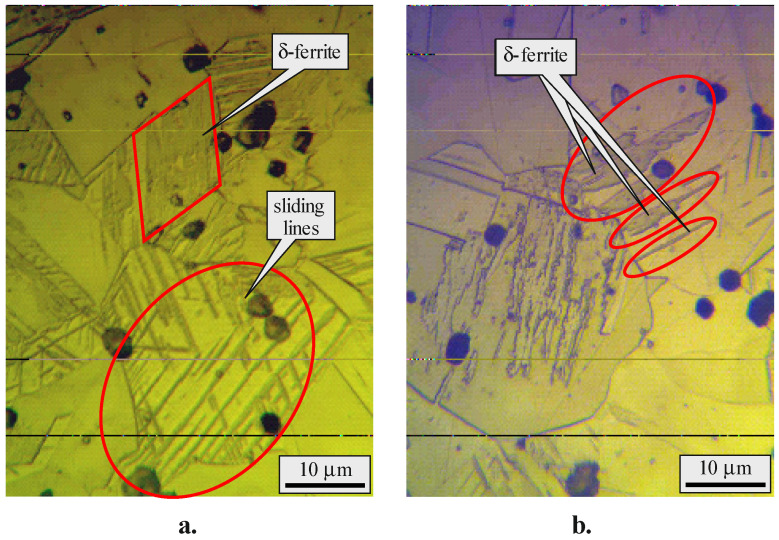
Initial microstructure of AISI 304 steel, magnification ×1000: (**a**) in the subsurface layers; (**b**) in the core.

The microstructure in the cross-section area of the cylindrical bar in its as-received state was observed via optical microscopy (OM, NEOPHOT 2) after polishing and etching of the specimen, using “royal water”—a mixture of nitric acid and hydrochloric acid in a molar ratio of 1:3 (HNO_3_:HCl = 1:3). The microstructure was characterised by structural inhomogeneity (Figure 3). Zones with sliding stripes, zones with equiaxed austenitic grains with an average size of 35 µm, twins, and sliding zones in the grains themselves were observed. Residual δ-ferrite was observed in and around the sliding zones. These areas likely have an increased local chromium content. The presence of *M*_23_*C*_6_ carbide was observed in these areas. The metal component M probably contains manganese and chromium. The presence of δ-ferrite reduces the corrosion resistance of the steel [31]. It should be noted that the resulting wells in the structure are a consequence of treating the sample with acid.

### 2.2. Diamond Burnishing Implementation

**Figure 4:**
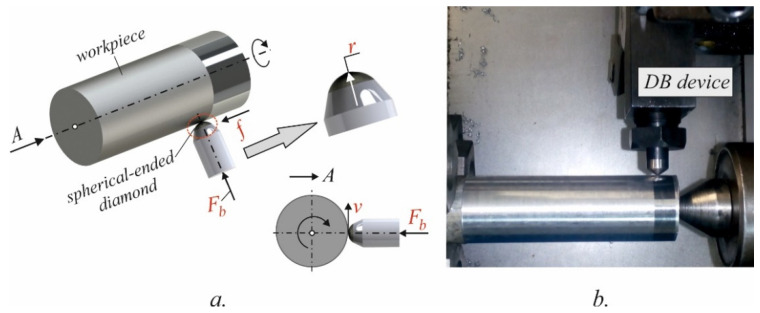
Diamond burnishing: (**a**) kinematic scheme; (**b**) implementation on CNC T200 lathe.

DB is a static mechanical surface treatment process based on severe plastic deformation due to the tangential sliding friction contact between the deforming diamond and the surface being treated. As a result, the surface integrity (SI) of the burnished component is improved, which reflects positively on the operating properties of the corresponding component; the fatigue strength, wear resistance and corrosion resistance are increased dramatically. DB kinematics is similar to those for turning (Figure 4a). The governing factors of the DB process are: sphere radius of the diamond insert r, mm; burnishing force $F_{b},\;N$; feed rate f, mm/rev; burnishing velocity v, m/min, and the number of passes n.

In order to study the process and its influence on SI, the diamond burnishing was performed on a CNC T200 lathe using a burnishing device with an elastic fixation of the deforming polycrystalline diamond insert (Figure 4b). A lubricant cooler Hacut 795-H was used. Turning as pre-machining and DB were carried out in a single clamping process to minimise the concentric run-out in burnishing. The turning was conducted along the full length of each specimen, while the treated length through slide burnishing with one combination of governing factors was 20 mm. Thus, for a group of experimental points (combinations of governing factors) the same initial average roughness before DB was ensured.

To evaluate the effect of DB on the characteristics of surface integrity (SI) of AISI 304 steel, a parametric study was carried out using the one-factor-at-a-time method. The rotating bending fatigue strength strongly depends on the severe plastic deformation of the surface and subsurface layers [33]. Residual compressive stresses are introduced, and the surface micro-hardness increases significantly due to strain hardening. A correlation exists between the surface micro-hardness and the fatigue strength of the diamond-burnished component. However, it should be noted that the relationship is not synonymous because an excessively large surface micro-hardness is associated with a significant increase in the density of the dislocations in the surface layer which leads to the introduction of microdefects. On the other hand, the expected effect of any burnishing process is to achieve minimal roughness, which is also a prerequisite for increasing rotating bending fatigue strength. Based on the above, two SI characteristics were controlled in the parametric study, the roughness parameter *R_a_* and surface micro-hardness.

### 2.3. SI Characteristics Measurement

#### 2.3.1. Roughness 2D Parameter $R_{a}$

The average 2D roughness parameter $R_{a}$ was measured using a Mitutoyo Surftest SJ-210 surface roughness tester. The final roughness value $R_{a}$ for each specimen was obtained as the arithmetic mean of five measurements of five generatrixes at a 72° angle.

#### 2.3.2. Micro-Hardness

The $HV_{0.05}$ surface micro-hardness measurements were made using a ZHVµ Zwick/Roell micro-hardness tester with automated processing of the measurement results, using a 0.05 kgf load and a 10 s holding time. Twenty measurements were made for each specimen. The final value of the surface micro-hardness corresponded to the grouping centre. In addition, the depth distribution of the microhardness was measured using a 0.025 kgf load and a 10 s holding time.

#### 2.3.3. Residual Stresses

The residual stresses were measured using X-ray diffraction residual stress technique [34]. A Bruker D8 ADVANCE diffractometer with a pin-hole collimator and a primary beam measuring 1 × 1 mm was used. The X-ray tube’s mode of operation (high voltage/current) was 30 kV/40 mA. The $\sin^{2}\psi$ method with a least-squares fitting procedure evaluated the residual stresses. From the phase analysis results, it was found that the change in the parameters of the DB process leads to different predominant phases in the surface and subsurface layer in the samples made of AISI 304L austenitic steel, which may be either *α-Fe* or *γ-Fe*.

The measured diffraction profile of the *α-Fe* {211} plane (of the *γ-Fe* {220} plane, respectively) has its maximum at $2\theta \approx 156.1^{{}^{\circ}}$ (at $2\theta \approx 128.8^{{}^{\circ}}$ for *γ-Fe*, respectively) for the filtered *VKα* radiation used. Diffraction profiles were determined using the Pearson VII method [35], and the lattice deformations were calculated. For the generalized Hooke’s law, the Winholtz and Cohen method with X-ray elastic constants $s_{1}=-1.271\;\mathrm{TP}a^{-1}$ and $\frac{1}{2}s_{2}=5.811\;\mathrm{TP}a^{-1}$ ($s_{1}=-1.352\;\mathrm{TP}a^{-1}$ and $\frac{1}{2}s_{2}=6.182\;\mathrm{TP}a^{-1}$ for *γ-Fe*, respectively) were applied. The parameters used in the X-ray experiment were a *2*θ range of $146^{{}^{\circ}}–161^{{}^{\circ}}$ ($124^{{}^{\circ}}–134^{{}^{\circ}}$ for γ-Fe, respectively), 2θ step of $0.5^{{}^{\circ}}$, and a tilt defined by $\sin^{2}\psi$ = 0, 0.1, 0.2, 0.3, 0.4, 0.5 for both positive and negative *ψ* angle values. The effective penetration depth of the *CrKα* radiation was approximately 6−7 μm (5 ÷ 6 μm for *γ-Fe*, respectively).

The stress gradient under the specimen’s surface, was analyzed by gradually removing material surface layers by electrolytic polishing. An ATM Kristall 650 Electrolytic Polisher with electrolyte based on a solution of perchloric acid and ethanol was used to remove of these layers.

### 2.4. Fatigue Tests 

#### 2.4.1. Hourglass-Shaped Fatigue Specimen Preparation

**Figure 5:**
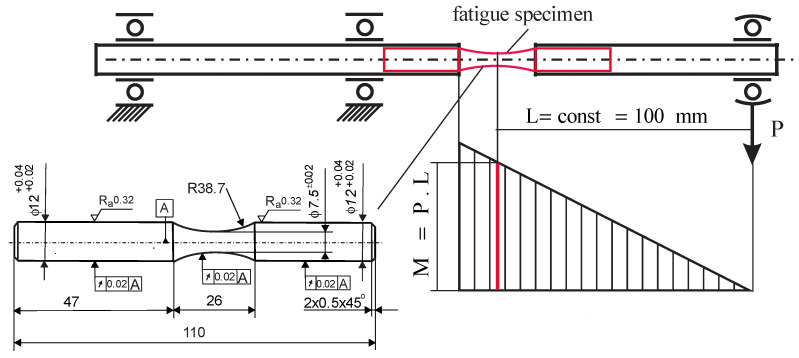
Specimen geometry (the sizes are in mm) and rotating bending fatigue test scheme.

Four groups of hourglass-shaped fatigue specimens with minimum diameters of 7.5 mm and lengths of 110 mm were manufactured on a CNC T200 lathe. Figure 5 shows the detailed geometry (according to the UBM testing machine’s requirements) of hourglass-shaped fatigue specimens. The first group was treated by cutting only, with the last pass consisting of fine turning in order to obtain the minimum possible roughness. Afterwards the specimens were polished to meet the roughness requirements (see Figure 5). The specimens from the other three groups were subjected to DB with differing process parameters. The second group was treated with parameters that provided minimum roughness (so-called smoothing DB); the third and fourth groups were diamond burnished according to parameters that provided the maximum surface micro-hardness (hardening DB). Five passes were used for the fourth group and one pass was used for the first three groups. The material was as received. The lubricant-cooler Hacut 795-H was used for both turning and DB.

#### 2.4.2. Experiment Conditions

Bending fatigue tests were conducted on a UBM testing machine. The scheme is shown in Figure 5. The loading frequency was 50 Hz in air. The accuracy of counting the number of cycles to fatigue failure is 100 cycles. The rotating load magnitude (the bending moment) was controlled via a lever system. The stress amplitude is calculated by the formula:

(1) $$\sigma =\frac{32PL}{\pi d_{min}^{3}}$$

where PL is the bending moment, $\frac{\pi d_{min}^{3}}{32}$ is the resistance moment of bending, *P* is the force see (Figure 5) that is set by the level system, *L* is the length of the cantilever part, $d_{min}$ is the minimum diameter of the sample. The accuracy of setting the force *P* is 1 N and of the length *L*—0.1 mm. Thus, the maximum error Δ of the stress amplitude σ is obtained from (1) after substituting *P =* 1 N, *L =* 100.1 mm, and the minimum diameter of the sample is 7.48 mm: i.e., Δ = 2.44 MPa.

The mechanical system, consisting of the two spindles and the specimen, was subjected to rotating three-point bending. The specimen, considered in isolation from the mechanical system, is subjected to cantilever rotating bending. Due to the rotation, the cycle asymmetry factor (of the stresses caused by the load from the testing machine alone) is *R =* −1. For each experimental point (i.e., stress amplitude), one specimen was tested in order to obtain an S–N curve, because the graphical visualization was made in a two-logarithmic coordinate system. Each specimen was tested to complete destruction. The exceptions were samples that reached $10^{7}$–cycle fatigue strength (i.e., the fatigue limit), after which the test was terminated. The S–N curves were built in a double-logarithmic coordinate system. The experiment aimed to quantify fatigue behaviour improvement as a percentage due to DB implementation.

#### 2.4.3. Fatigue Strength Improvement Due to DB

The fatigue strength improvement (FSI) of chromium-nickel AISI 304 austenitic stainless steel due to DB in comparison with the basic material was calculated via the formula

(2) $$FSI=\frac{\sigma_{i}^{DB}-\sigma_{i}}{\sigma_{i}}\times 100\%,$$

where $\sigma_{i}^{DB}$ is the $N_{i}$–cycle fatigue strength obtained due to DB and $\sigma_{i}$ is the $N_{i}$–cycle fatigue strength taken from the reference condition S–N curve (obtained for the specimens treated by turning and polishing).

## 3. Results

### 3.1. Parametric Study

The mean value of the initial roughness parameter $R_{a}$ (after fine turning and before DB) is in the range (0.423–0.685)μm for all samples.

#### 3.1.1. Effect of the Radius and Burnishing Force

**Figure 6:**
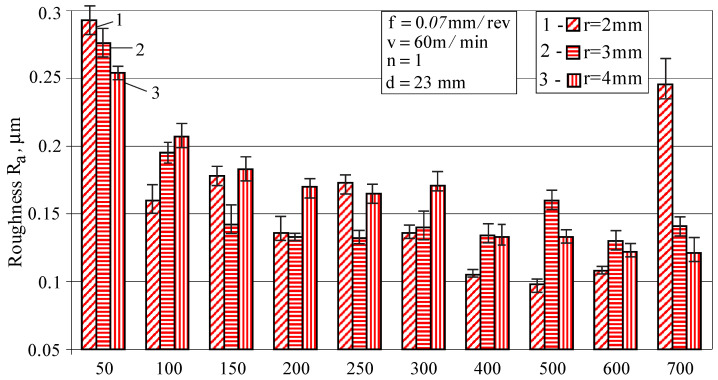
Effect of the radius and burnishing force on the roughness parameter $R_{a}$.

Both factors affect the roughness parameter $R_{a}$, the more significant of which is the burnishing force (Figure 6). In the range (200–600)N of force change, all radius values provide relatively low values of $R_{a}$. The minimum values of $R_{a}$ are provided by combinations of the radius r=2 mm with values of burnishing force in the range (400–600)N. A subsequent increase in force in combination with r=2 mm sharply deteriorates the roughness in terms of the parameter $R_{a}$.

**Figure 7:**
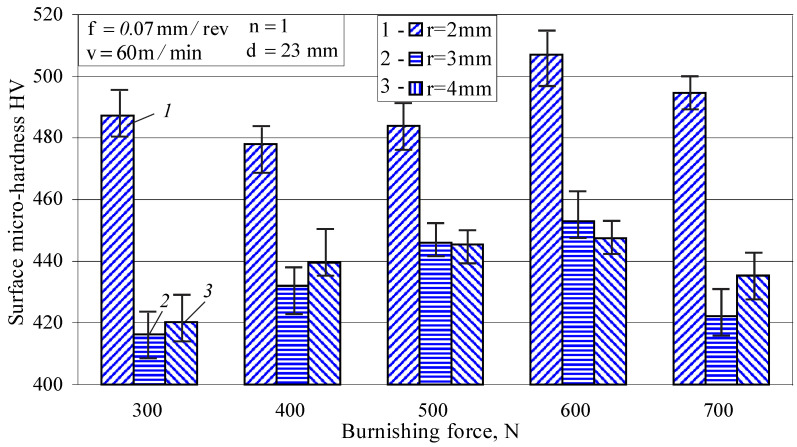
Effect of the radius and burnishing force on the surface micro-hardness.

Evidently, the radius has a greater effect on the surface micro-hardness than the burnishing force (Figure 7). The highest micro-hardness is obtained using r=2 mm for all values of the burnishing force. The other two values of the radius, 3 and 4 mm, lead to close values of the micro-hardness for all of the applied values of the burnishing force. In order to obtain the maximum surface micro-hardness, the burnishing force should not exceed 600 N. Increasing the burnishing force leads to an increase in the equivalent plastic strain in the contact zone with the deforming diamond insert. The combination of radius and burnishing force, which leads to the maximum equivalent plastic strain of the specimen surface, provides the maximum surface micro-hardness. A further increase in the burnishing force increases the depth of the plastic area and shifts the maximum equivalent strain to points below the surface layer. This explains the decrease in surface micro-hardness when the burnishing force increases above 600 N.

Based on the above, it can be concluded that a radius r=2 mm and a burnishing force $F_{b}=600\;N$ provides low roughness $\left(R_{a}=0.108\;\mu m\right)$ and high surface micro-hardness (507 HV).

#### 3.1.2. Effect of the Feed Rate

**Figure 8:**
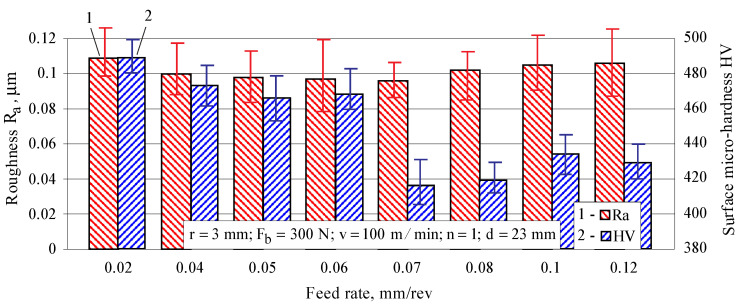
Effect of the feed rate on the roughness parameter $R_{a}$ and surface micro-hardness.

The effect of the feed rate on the roughness parameter $R_{a}$ and surface micro-hardness is shown in Figure 8. The feed rate has little effect on the roughness parameter $R_{a}$. Conversely, the feed rate strongly influences the micro-hardness. The lowest feed rate leads to the greatest micro-hardness due to the so-called overlapping effect [32]. The minimum values of $R_{a}$ and the surface micro-hardness are provided simultaneously by the same feed rate value: f=0.07 mm/rev.

#### 3.1.3. Effect of the Burnishing Velocity

**Figure 9:**
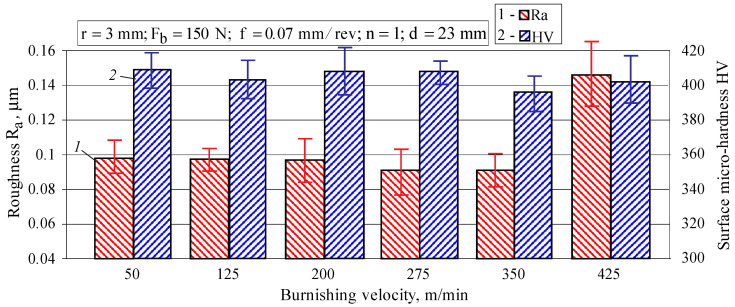
Effect of the burnishing velocity on the roughness parameter $R_{a}$ and surface micro-hardness.

The effect of the burnishing velocity on the roughness parameter $R_{a}$ and surface micro-hardness is shown in Figure 9. The parameter $R_{a}$ is weakly influenced by the burnishing velocity because in the velocity interval (50–350) m/min the values of $R_{a}$ are practically constant, with a slight tendency to decrease. Some deterioration is observed at v=425 m/min. It should be noted that high speeds (v>150 m/min) are not recommended as they lead to intense diamond wear and local damage to the treated surface due to a significant increase in the heat generated.

In the (50–425) m/min velocity range, the measured micro-hardness varies from 396 to 409 HV. It can be concluded that the burnishing velocity has a small effect on the resulting micro-hardness for AISI 304 steel, showing a slight tendency to reduce the surface micro-hardness when the burnishing velocity increases. The high speeds (v>200 m/min) lead to several undesired effects, such as relatively high temperature in the contact area, accelerated diamond wear, softening effect, and others. Thus, in practice, the speed rarely exceeds 150 m/min. Therefore, it can be assumed that the burnishing velocity practically does not affect the SI of AISI 304 steel.

#### 3.1.4. Effect of Number of Passes

**Figure 10:**
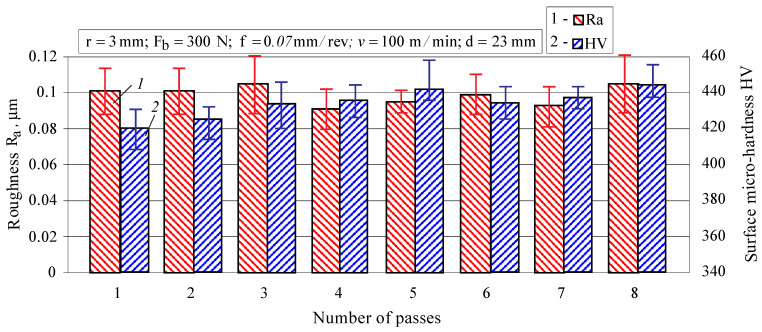
Effect of the number of passes on the roughness parameter $R_{a}$ and surface micro-hardness.

The effect of the number of passes on the roughness parameter $R_{a}$ and surface micro-hardness is shown in Figure 10.

The parameter $R_{a}$ is weakly influenced by the number of passes, and its minimum values are obtained at four passes. A steady trend is observed with an increase in the micro-hardness when the number of passes increases from one to five. A similar trend, but less pronounced, is observed with a further increase in passes to eight. DB causes cyclic loading in the vicinity of each point from the surface layer. Probably at *n =* 5, a stabilised cycle is reached, and the micro-hardness changes slightly for a further increase in the number of passes. Detailed information on achieving a stabilised cycle in the DB process is given in [36].

#### 3.1.5. Selection of Values of the Governing Factors, Ensuring Minimal Roughness

In this study, the term “minimal roughness” is understood to mean the resulting roughness (one of the three surface texture characteristics), for which the height parameter $R_{a}$ satisfies the condition $R_{a}\leq 0.1\;\mu m$. Based on the one-factor-at-a-time results (see Figure 6, Figure 8 and Figure 9), the following values of the main governing factors were chosen: r=3mm, $F_{b}=300\;N$, f=0.07 mm/rev and v=100 m/min. Additional tests with these values showed that the roughness parameter $R_{a}$ of the diamond burnished surfaces varies around 0.1 μm.

#### 3.1.6. Optimization under Maximum Surface Micro-Hardness Criterion

**Figure 11:**
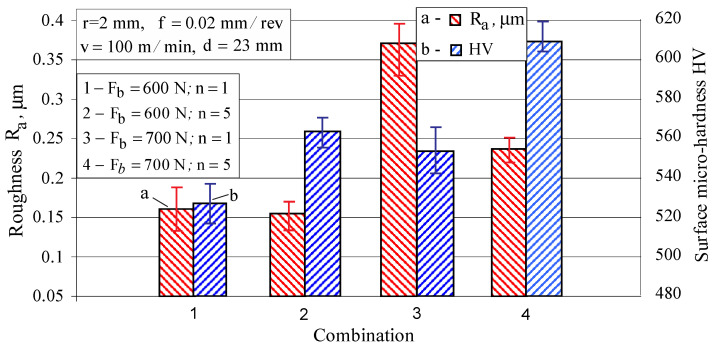
Some combination providing high micro-hardness.

Based on a one-factor-at-a-time study, it can be concluded that from the main parameters of the DB process the radius of the deforming diamond, burnishing force and feed rate significantly affect the micro-hardness. The effect of the number of passes is also noticeable. Figure 11 shows the impact of some combinations of these parameters. The combination of minimum radius, maximum force, minimum feed rate and number of passes *n =* 5 lead to maximum surface micro-hardness: the initial micro-hardness after turning and fine turning from 366 HV increases to 609 HV through DB, i.e., an increase of 66.4%.

### 3.2. Evaluation of the Strain Induced Martensite

As DB causes severe plastic deformation of the surface layer, part of the austenite in this layer (and the adjacent subsurface layers) is transformed into α’-martensite. The greater the plastic deformation, the greater the amount of martensite. The percentage of martensite in the surface and subsurface layers is necessary information to determine the residual stresses introduced by DB.

To determine the ratio between the austenitic and martensitic phases, a DIFFRAC.DQuant V1.5 specialized software developed by BRUKER company (Billerica, MA, USA) was used [37]. The phase distribution was determined, taking into account the ratio between the areas of the austenitic peaks (200) and (220) to the areas of the martensitic peaks (200) and (211). The relative intensity of the individual peaks was also taken into account when calculating the percentage ratio between the two phases. The accuracy of the phase distribution measurement depends on the accuracy of the obtained diffraction pattern and increases when the measurement time (time/step) is longer and the increment step of the 2θ angle (°/step) is smaller. For greater accuracy against the background of the diffraction pattern and the obtained peaks, the measurement was repeated in the “auto replay” mode until a “smoothed” diffraction pattern was obtained.

**Figure 12:**
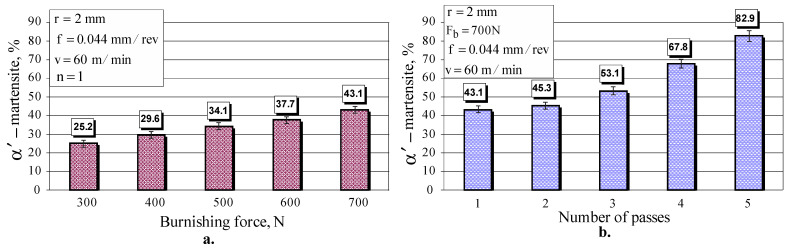
Effect of: (**a**) burnishing force, and (**b**) number of passes, on strain-induced martensite in the surface layer.

Two cylindrical specimens, both with five sections, a diameter of 23 mm and a length of 20 mm, were subjected to DB on a conventional C11 lathe using the following parameters: diamond radius r=2 mm, burnishing velocity v=60 m/min and feed rate f=0.044 mm (the lowest feed rate that a C11 lathe can provide) The five sections of the first sample were processed using one pass (n=1), and for each section, a different burning force, respectively, 300, 400, 500, 600 and 700 N, was used. The second specimen was subjected to DB, and each section was processed with a different number of passes: 1, 2, 3, 4, and 5. It was established (see Figure 10 and Figure 11) that five passes (n=5) ensured maximum micro-hardness. Figure 12 shows the influence of the burnishing force and number of passes on the percentage of strain-induced *α*’-martensite introduced into the surface layer. As the burnishing force increases, the percentage of martensite increases in an approximately linear fashion. The number of passes is the more significant of the two factors. Increasing the number of passes leads to an increase in martensite by nonlinear law. Five-pass DB doubles the amount of martensite due to the generation of greater plastic deformation compared to single-pass DB.

**Figure 13:**
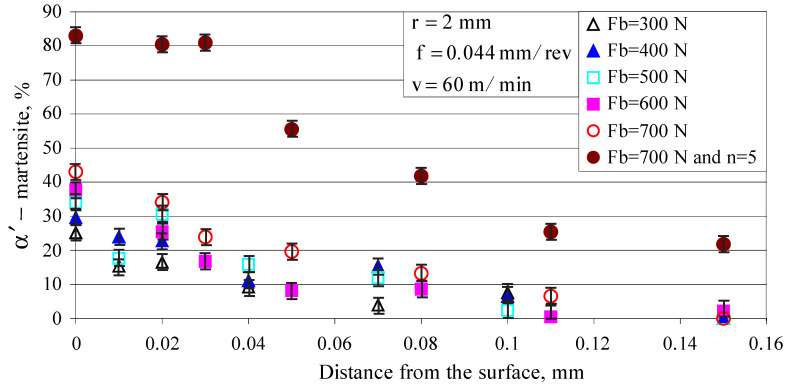
Distribution of the strain-induced martensite in a depth.

Figure 13 shows the depth distribution of the strain-induced martensite. A single-pass DB (using the above parameters) causes martensitic transformation to a depth of 0.15 mm; the depth is significantly increased if multi-pass DB was applied.

Of interest is the percentage of strain-induced *α*’-martensite, measured on the surface (in the area around the smallest diameter) of the diamond burnished fatigue specimens before the fatigue tests and introduced via different types of DB. The average value of the amount of strain-induced α’-martensite is as follows: 53.7%, 87.5% and 98.6%, for smoothing DB, single-pass hardening DB and five-pass hardening DB, respectively. The measured values are significantly higher than those of the cylindrical specimens (see Figure 12), as the fatigue specimens were diamond burnished using a minimum feed rate—*f* = 0.02 mm/rev. Since the α’-phase is harder, the micro-hardness obtained is a maximal (see Figure 8) when *f =* 0.02 mm/rev. Higher micro-hardness provides greater fatigue strength.

### 3.3. Residual Stresses

The depth distribution of the residual stresses introduced by turning and DB was measured via XRD on cylindrical specimens with a diameter of 23 mm and a length of 20 mm, using the information obtained from Section 3.2 on surface amount and strain-induced $\alpha^{\prime }—$ martensite depth distribution. Four samples were processed on conventional C11 lathe in a manner as follows: (1) only by cutting: turning rough and fine, and polishing; (2) fine turning and single-pass DB, implemented via parameters to provide minimum roughness, namely: *r =* 3 mm, *F_b_ =* 300 N, *f =* 0.07 mm/rev and v = 60 m/min (smoothing DB); (3) as (2), but with parameters chosen to provide micro-hardness close to the maximum: *r =* 2 mm, *F_b_ =* 300 N, *f =* 0.044 mm/rev and *v =* 60 m/min (hardening single-pass DB); and (4) as (3), but with hardening five-pass DB. For the first two samples, the measurement was performed on the γ-phase, and for the last two samples on the two phases, *α’* and *γ*, given the significant amount of the strain-induced α’-martensite in the surface and subsurface layers (see Figure 13).

**Figure 14:**
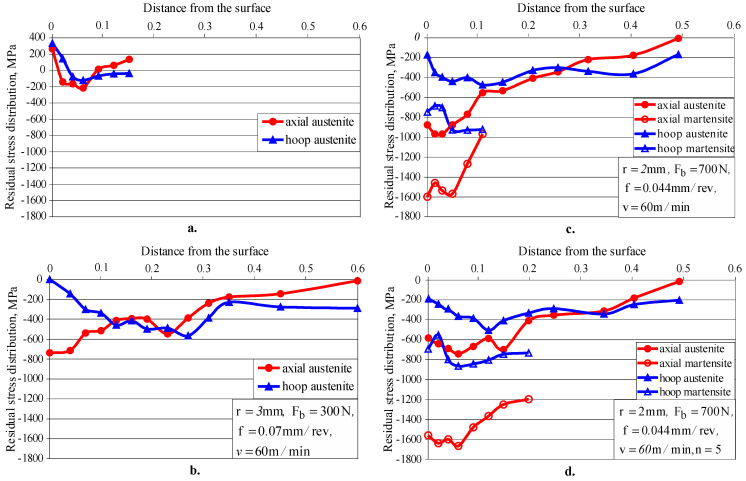
Residual stress distribution: (**a**) processed by turning only (austenitic phase); (**b**) smoothing single-pass DB (austenitic phase); (**c**) hardening single-pass DB (austenitic and martensitic phases); (**d**) hardening five-pass DB (austenitic and martensitic phases).

Figure 14 shows the residual stress distribution for the four samples. For each experimental point (distance from the surface), 11 measurements were made, on the basis of which an approximation line was constructed. The maximum deviations Δ*σ* were calculated according to the Pearson VII method. For the austenitic phase, the deviations vary from 17 MPa to 71.1 MPa, and for the martensitic phase—from 23 MPa to 50.4 MPa. Larger values of deviations are obtained for smaller absolute values of residual stresses.

The turning introduces residual tensile stresses in the surface layer and low residual compressive stresses at depths up to 0.1 mm (Figure 14a). The smoothing single-pass DB introduces significant compressive residual stresses at a depth from the surface layer greater than 0.6 mm (Figure 14b). As the amount of strain-induced martensite is less than 10% at depths greater than 0.04 mm (see Figure 13, *F_b_ =* 300 N), the residual stress measurement was performed on the 220 *γ*-phase only. Due to the higher burnishing force and lower feed rate, the hardening single-pass DB introduces significantly higher (absolute value) residual stresses than the smoothing DB (Figure 14c), as measured in the 220 *γ*-phase. The residual stresses for the 211 *α*’-phase (martensite) were measured at a depth of up to 0.1 mm, since in the deeper layers the martensite content is below 10%. As expected, the residual stresses measured for the martensitic phase are significantly higher in absolute value than those for the austenitic phase, as the martensite is significantly harder. The application of five-pass hardening DB changes the picture of residual stresses (Figure 14d) compared to those introduced by single-pass hardening DB. The hoop stresses measured for both phases remained virtually unchanged, while the axial stresses measured for the austenitic phase decreased significantly in absolute value. Conversely, the axial residual stresses measured for the martensitic phase to a depth of 0.2 mm increased significantly in absolute value.

**Figure 15:**
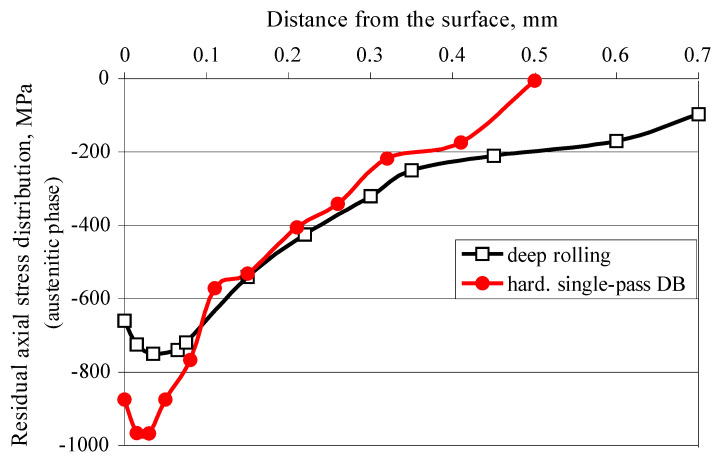
A comparison of the axial residual stresses measured for the austenite phase and obtained via deep rolling [26] and DB.

Figure 15 shows a comparison of the axial residual stresses introduced by hardening single-pass DB and measured for the austenitic phase with those reported by Juijerm and Altenberger [26] and introduced by Ecoroll’s deep rolling process. The axial residual stresses introduced by DB are significantly larger in absolute value in the surface layer and in the subsurface layers, at a depth of approximately 0.1 mm. In the interval 0.1–0.4 mm, the two groups of residual stresses differ slightly from each other, with a slight advantage of those introduced by deep rolling. However, the deep rolling process achieves a greater depth of the compressive zone.

### 3.4. Rotating Bending Fatigue Results

**Figure 16:**
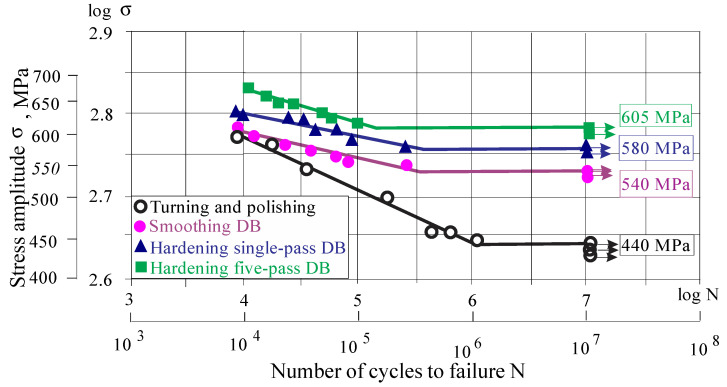
S–N curves.

According to Section 3.1.6, the third and fourth groups of hourglass-shaped fatigue specimens were diamond burnished according to the following parameters: *r =* 2 mm, *F_b_ =* 700 N, *f =* 0.02 mm/rev and *v =* 100 m/min, with one (hardening single-pass DB) and five passes (hardening five-passes DB). The second group of specimens was subjected to smoothing DB: *r =* 3 mm, *F_b_ =* 300 N, *f =* 0.07 mm/rev and *v* = 100 m/min (see Section 3.1.5). The S–N curves in a double-logarithmic coordinate system are shown in Figure 16.

Compared with the reference condition (turning and polishing), the smoothing DB increases the fatigue limit (10^7^ fatigue strength) from 440 MPa to 540 MPa. As expected, the effect of applying hardening DB is significantly greater than that of smoothing DB. Single-pass DB increases the fatigue limit to 580 MPa and applying five-pass DB multiplies the effect up to 605 MPa. It should be noted that all three DB implementation options are less effective in the field of low-cycle fatigue (LCF).

## 4. Discussion

**Figure 17:**
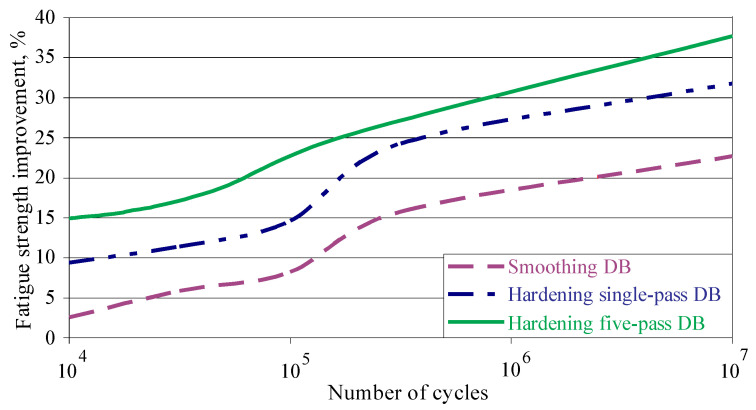
Fatigue strength improvement.

FSI due to DB was calculated using formula (2) and Figure 16. Figure 17 illustrates FSI in both low-cycle fatigue (LCF) and high-cycle fatigue, depending on the number of cycles to fatigue failure. The effect of DB increases with decreasing the bending stress amplitude. In other words, FSI is the smallest in the LCF field and the largest for the fatigue limit. The lower FSI in the LCF field can be explained by the residual stress relaxation, which is based on the evolution of the material structure, due to the fatigue cyclic loading and heat generated by internal friction. Zhuang and Halford [38] studied the relaxation of residual stresses introduced via different surface treatments of Inconel 718 due to cyclic load, whose amplitude is less than the material yield limit. They established that the relaxation rate increased when the internal stress amplitude increases. In addition, rapid relaxation was observed during the first load cycles, after which the relaxation rate decreased.

**Figure 18:**
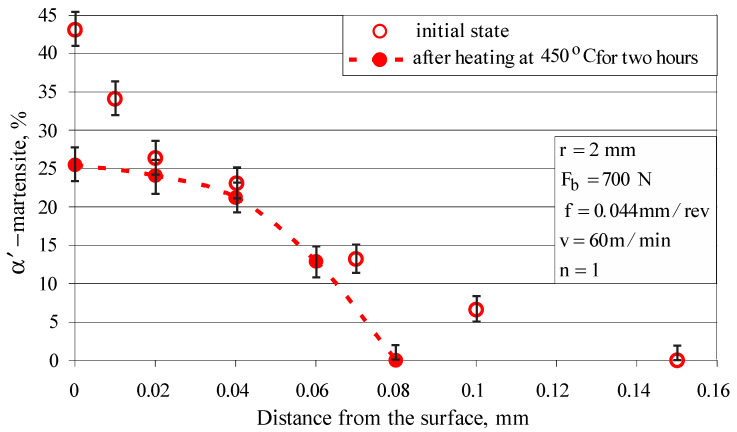
Reverse martensitic transformation due to heating.

**Figure 19:**
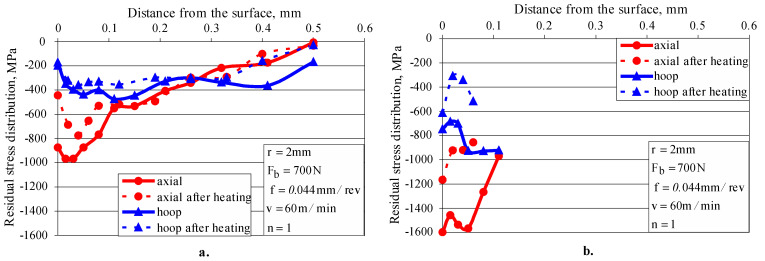
Residual stress relaxation due to heating (at $450{\;}^{{}^{\circ}}C$ for two hours): (**a**) austenitic phase; (**b**) martensitic phase.

To experimentally determine the relaxation of the residual stresses introduced via DB only from thermal action, a cylindrical specimen with a diameter of 23 mm was diamond burnished (r=2 mm, $F_{b}=700\;N$, f=0.044 mm/rev, v=100 m/min and n=1) and then heated at $450{\;}^{{}^{\circ}}C$ for two hours. Figure 18 and Figure 19 show the reverse martensitic transformation $\left(\alpha^{\prime }\to \gamma \right)$ and residual stress relaxation, respectively, a consequence only of elevated temperature ($450{\;}^{{}^{\circ}}C$), but less than that of recrystallization for the investigated AISI 304 steel. The martensitic (harder) phase is reduced almost twice on the surface and is preserved (although significantly reduced) at a depth of less than 0.08 mm (Figure 18). As a result, the surface micro-hardness and compressive residual stresses decrease, which leads to a decrease in fatigue strength. Although the temperature is lower than the recrystallization temperature, the deformed crystal lattice regains its original correct shape, but the slippage of the crystallites in the respective crystallographic planes is preserved. This effect explains the relaxation of the residual stresses due only to a temperature close to (but less than) the recrystallization temperature (Figure 19). In fact, due to the large hysteresis in the LCF field, where the values of the bending stresses significantly exceed the material yield limit, the temperature in the sample exceeds the recrystallization temperature. This, in combination with the Bauschinger effect [38], quickly reduces the residual compressive stresses to zero when the bending stress significantly exceeds the yield limit.

FSI is significantly increased in the HCF field for the following reasons: (1) The bending stresses are lower, as a result of which the relaxation of the compressive residual stresses is less pronounced; (2) DB causes strain hardening of the surface and subsurface layers, i.e., the yield limit of these layers increases, and as a result, the bending stresses are in the elastic field; (3) Less pronounced reverse martensitic transformation $\alpha^{\prime }\to \gamma$, due to which the surface micro-hardness remains relatively high.

It has been proven [39] that the destruction of diamond burnished specimens due to fatigue starts from fatigue cracks that originate at the boundary between the affected layer and bulk material. Therefore, the residual stresses and surface micro-hardness play a crucial role for FSI compared to surface roughness. The above explains why smoothing DB provides the lowest FSI. The smoothing DB ensures the smallest surface micro-hardness due to the relatively small amount of strain-induced $\alpha^{\prime }—$ martensite in the surface layer (see Figure 8). The dominant softer austenitic phase is reflected in the smaller absolute values of the compressive residual stresses compared to hardening DB (Figure 14b). As a result of hardening DB, the harder martensitic phase dominates in the surface and subsurface layers. The combination of high burnishing force (700 N) and small radius (2 mm) causes significant plastic deformation. Therefore, the residual stresses measured for the austenitic phase are significantly higher in absolute value than those introduced via smoothing DB. It should be noted that the residual stresses measured for the martensitic phase, which are significantly higher than those measured for the austenitic phase, are crucial for the fatigue behaviour of the specimens processed via hardening DB.

Five-pass hardening DB significantly increases the amount of strain-induced $\alpha^{\prime }—$martensite both on the surface and within the material bulk (see Figure 13). As a result, the surface micro-hardness and the depth of the compressive field measured for the martensitic phase are significantly increased (Figure 14d). This explains the better FSI result achieved by five-pass DB compared to hardening single-pass DB (Figure 17).

Figure 17 shows the great potential of DB to increase the fatigue limit of AISI 304 steel. Depending on the type of DB, this increase is between 23% and 38%. For comparison, Hoshiyama et al. [29] reported an increase of approximately 15% achieved by low-temperature nitriding. Juijerm and Altenberger [26] reported an increase of approximately 18% at 10^6^ cycles fatigue strength using deep rolling process, while for the same number of cycles fatigue strength the hardening five-pass DB approximately achieved a 31% increase.

## 5. Conclusions

This paper aims to quantify the effect of DB on rotating bending fatigue strength of AISI 304 chromium-nickel austenitic stainless steel, based on a comparison with the effect achieved by fine turning and polishing as a reference condition. An experimental approach was used, including a parametric DB process study, an assessment of the SI obtained after different DB processes, an evaluation of the strain-induced $\alpha^{\prime }—$martensite and rotating bending fatigue tests. As a result of this work, the major new findings concerning the mechanism of FSI were:

(1) The values of the parameters of DB of AISI 304 steel were found, through which smoothing and hardening DB processes are realised;

(2) The initial state characteristics of SI (roughness, micro-hardness, residual stresses, $\alpha^{\prime }—$martensite), obtained via smoothing and hardening DB processes, are quantified;

(3) For the first time, the possibilities of DB were discovered as a mechanical surface treatment process for inducing $\alpha^{\prime }-$ martensite in surface and subsurface layers of AISI 304 steel. It has been found that an increase in the percentage of the martensitic phase leads to an increase in fatigue strength;

(4) Different types of DB processes increase the fatigue strength of AISI 304 steel by 23–38%. Therefore, in terms of cost/quality ratio, DB is superior to low-temperature nitriding when the goal is to increase the fatigue limit of AISI 304 steel, and the requirements to increase wear resistance are secondary.

## Figures and Tables

**Table 1:** Chemical composition in weight percentages (wt%) of the tested AISI 304 steel.

Fe	C	Si	Mn	P	S	Cr	Ni	Mo	Cu	Nb	Ti	V	W	Others
71.5	0.036	0.193	1.52	0.03	0.026	17.7	8.3	0.182	0.25	0.042	0.003	0.07	0.05	balance

**Table 2:** Main mechanical properties at room temperature of the studied AISI 304 steel (as-received).

Young’s Modulus, GPa	Yield Limit, MPa	Ultimate Tensile Strength, MPa	Elongation, %	Percentage Reduction of Area, %
200	432	734	41	68.8

## Data Availability

Not applicable.
